# Piezo1-related physiological and pathological processes in glioblastoma

**DOI:** 10.3389/fcell.2025.1536320

**Published:** 2025-02-21

**Authors:** Weijia Fu, Xue Hou, Lijuan Ding, Jiaying Wei, Wei Hou

**Affiliations:** ^1^ Department of Radiation Oncology and Therapy, The First Hospital of Jilin University, Changchun, China; ^2^ Jilin Provincial Key Laboratory of Radiation Oncology and Therapy, The First Hospital of Jilin University, Changchun, China; ^3^ NHC Key Laboratory of Radiobiology, School of Public Health, Jilin University, Changchun, China

**Keywords:** glioblastoma, piezo1, biomechanical, targeted therapy, mechanomechanical

## Abstract

**Introduction:**

Glioblastoma (GBM) is the most malignant of the astrocytomas, primarily involving the cerebral hemispheres and cerebral cortex. It is one of the fatal refractory solid tumors with a 5-year survival rate of only 5% in adults. Cells in biological tissues are subjected to mechanical forces, including hydrostatic pressure, shear stress, compression and tension. Cells can convert mechanomechanical signals into biological or electrical signals, a process known as mechanical signaling. Piezo1 channels, members of the Piezo family of mechanosensitive ion channels, can be directly activated by mechanical stimuli alone, mediating mechanosensitive cation currents that activate subsequent signaling pathways. Studies have shown that Piezo1 is largely unexpressed in normal brain tissues but is expressed at high levels in glioblastoma and can significantly contribute to glioblastoma development and progression, but its role in the pathogenesis of glioblastoma remains unclear.

**Methods:**

We reviewed the relevant literature and data in six major databases including PubMed, EMBASE, CINAHL, Scopus, Web of Science and TCGA. Finally, a total of 126 papers were selected for review and analysis (Search terms include: glioblastoma, piezo1, biomechanical, targeted therapy, mechanomechanical, extracellular matrix, radiation therapy and more). The role of piezo1 in the development of glioblastoma was summarized.

**Results:**

Piezo1 affects several fundamental pathophysiological processes in glioblastoma, such as tissue sclerosis, angiogenesis, energy supply, and immune cell infiltration, and can be used as an indicator of malignancy and prognosis in patients with glioblastoma, as well as a therapeutic target to control tumor progression.

**Discussion:**

The pathological mechanism of piezo1 in glioblastoma is very complex, and the aberrant expression of piezo1 plays a very important role in the development of glioblastoma. Specific mechanistic studies focusing on Piezo1 will help us understand the mechanobiology of glioblastoma and help us develop new therapeutic approaches for glioblastoma patients.

## 1 Introduction

Glioblastoma (GBM) is the most malignant type of glioblastoma featuring fast proliferation, strong invasion, and poor prognosis (Peng et al., 2018). Patients with GBM usually show a variety of clinical manifestations that are caused by dysfunction of affected areas of the brain, such as lethargy, apathy, blindness, seizures, and language changes (McKinnon et al., 2021). Besides, it often grows in the brain parenchyma in an invasive manner (Lah et al., 2020).

Cells in biological tissues are subject to mechanical forces, including hydrostatic pressure, shear stress, compression, and tension (Humphrey et al., 2014). In biological systems, tissue stiffness varies considerably between organs and between healthy and diseased states of the same organ (Kai et al., 2016). In some regions, solid tumor tissue is harder than its untransformed counterpart, e.g., normal brain tissue is usually less than 100 Pa, whereas human low to high-grade glioblastomas (LGG and HGG) have progressively stiffer tissues ranging from 100 to 10^4^ Pa. This is due to the uncontrolled proliferation of cells in confined spaces, vascular hyper-permeability, insufficient lymphatic drainage, and increased deposition of extracellular matrix (ECM) protein deposition increases. Miroshnikova et al. used atomic force microscopy to quantify the hardness of non-tumor glial proliferation as well as glial cell tissue with different levels of malignant transformation in frozen human brain biopsies. The results showed that glial tissues have the lowest ECM hardness (Young’s modulus, E; 10–180 Pa), which increases progressively with the level of malignancy (50-1,400 Pa for LGGs and 70-13,500 Pa for GBM). NanoString nCounter gene expression analysis of prognostic predictor gene clusters from human GBM tissue biopsies (aggressiveness was categorized on a scale of 1–10, with 1 indicating the most aggressive) indicated that there was a significant correlation between ECM hardness and prognosis of glioblastomas, with higher hardness being associated with a worse prognosis (Miroshnikova et al., 2016). Chauvet et al. used intraoperative shear wave elastography to measure the elasticity of tumors and surrounding tissues in patients undergoing brain tumor resection. The study showed that the mean elasticity of meningiomas, low-grade gliomas, high-grade gliomas, and brain metastases was 33.1 ± 5.9 kPa (3.3 ± 0.6 m/s), 23.7 ± 4.9 kPa (2.8 ± 0.6 m/s), 11.4 ± 3.6 kPa (1.9 ± 0.6 m/s), and 16.7 ± 2.5 kPa (2.4 ± 0.4 m/s), respectively, demonstrating that the greater the tissue stiffness, the higher the malignancy., again demonstrating that the greater the tissue hardness, the higher the malignancy of the glioma (Chauvet et al., 2016). Increased tissue stiffness leads to tumor progression by modulating proliferation, invasion, apoptosis evasion, drug resistance, angiogenesis, metabolism, and growth-promoting signaling pathways (Northey et al., 2017; Oudin and Weaver, 2016).

Cells can convert mechanomechanical signals into biological or electrical signals, a process known as biomechanical signal transduction. Mechanical signal transduction pathways are often classified into three categories: cell membrane receptors and surface mechanosensitive ion channels, the cytoskeleton (stress fibers, microtubules, etc.), and the nucleoskeleton and cytoskeletal linker complexes (Chaudhuri et al., 2018). Mechanosensitive ion channels are protein pores embedded in the plasma membrane. When the channel is stimulated to open by a mechanical signal, ions enter the cell from the pore in the direction of the electrochemical gradient, without the need for energy from adenosine triphosphate (ATP) hydrolysis.

Piezo1 channels are a member of the Piezo family of mechanosensitive ion channels, the first mechanically gated cation channel family identified and established in mammals, which can be directly activated by mechanical stimuli alone to mediate mechanosensitive cationic currents (Coste et al., 2012). Piezo1 is mainly found in tissue cells that are sensitive to mechanical tension stimuli, such as the lung, bladder, and skin, and is involved in the development of various tumors (Coste et al., 2010). Studies have shown that Piezo1 is largely unexpressed in normal brain tissue, but is expressed at high levels within glioblastomas. However, their role in the pathogenesis of GBM remains unclear. This paper aims to summarise the role of Piezo1 in the development of GBM and the role of Piezo1 in the treatment of GBM and to discuss the role of Piezo1 inhibitors in the treatment of GBM.

## 2 Piezo1

**FIGURE 1:**
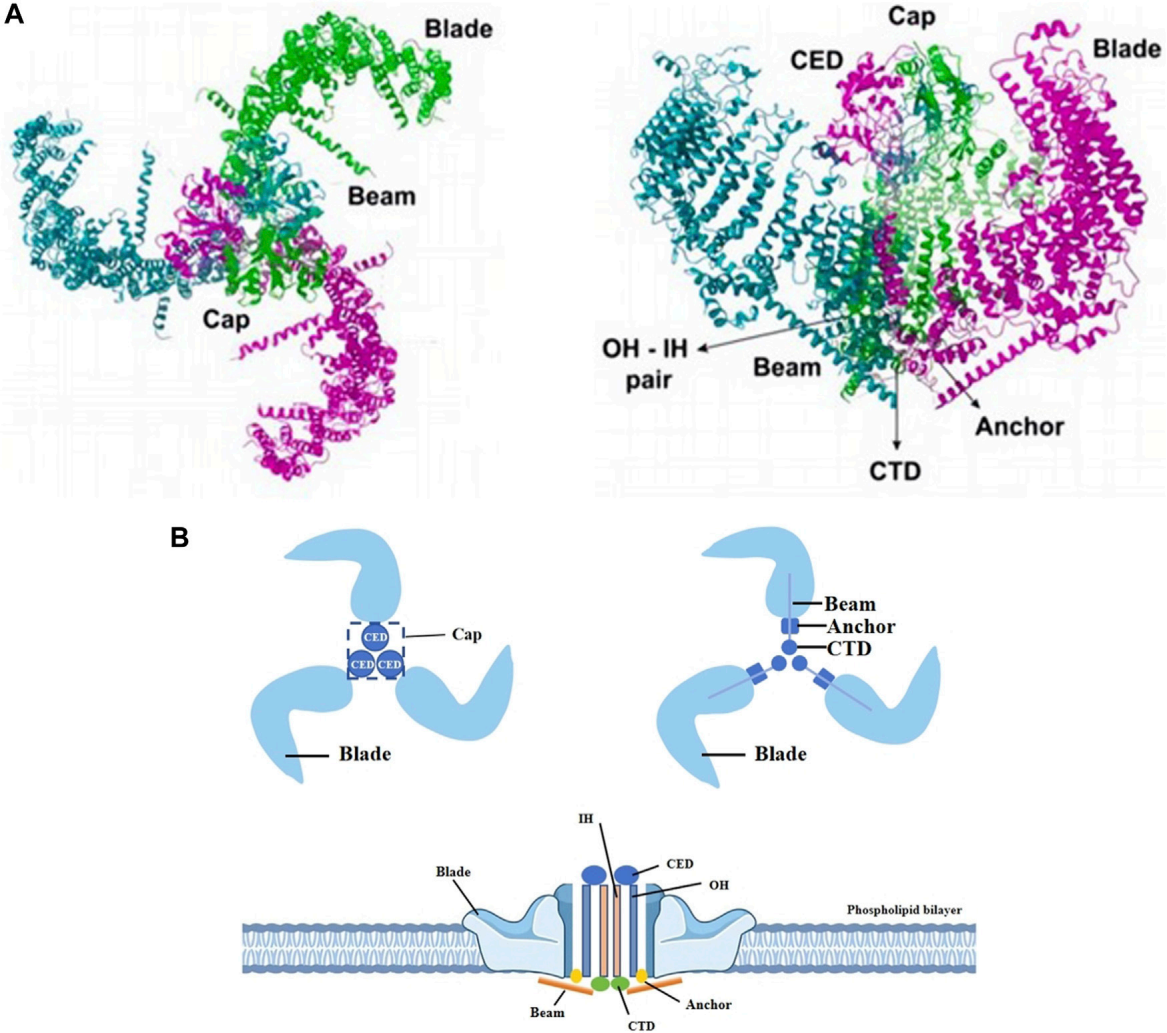
Different views of piezo1. **(A)** The Piezo1 protein exhibits a propeller-like three-bladed structure, with the 38 transmembrane helices of the three subunits coming together in the membrane plane to form a signature nanobowl-like structure. Adapted from (Thien et al., 2024). **(B)** Two transmembrane helices (OH - IH), the extracellular C-terminal structural domain (CED) and the intracellular C-terminal structural domain (CTD) trimer form the transmembrane channel of Piezo1. The CED, located between the OH and the IH, is the tip of the ion channel, and cations enter the channel through the open window (Cap) located directly above the membrane. The IH constricts in the middle of the membrane and forms the neck of the channel. When an external mechanical force stimulates the membrane, the stress causes the Piezo1 channel to change from a closed state to an open state, which induces Ca^2+^ influx into the interior of the cell and triggers different signaling pathways to follow.

The Piezo1 gene is located on human chromosome 16p24.3 and consists of 51 exons, 2547 amino acids and 3 transcripts (Yu and Liao, 2021). In plan view, it looks like a propeller blade or a tricuspid with an ion-penetrating pore in the middle and a cap c-terminal extracellular structural domain (CED) at the top. When viewed from the side, the Piezo1 channels distributed across the cell membrane interact with the lipid membranes surrounding the channels to form a dome-like structure (Guo and MacKinnon, 2017; Lin et al., 2019; Yang et al., 2022) and use the aforementioned trimeric structure to mediate mechanical force transfer via a lever principle mechanism (Wang et al., 2018; Thien et al., 2024). The central ion-conducting pore and the three peripheral propeller-like blades are two important regions for the function of Piezo1 as a mechanosensitive channel. Two transmembrane (TM) helices, called outer helix-inner helix (OH - IH) pairs, the C-terminal extracellular structural domain (CED) and the intracellular C-terminal structural domain (CTD) trimer form the transmembrane channel of Piezo1. The CED is located on top of the central ion pore and has been shown to regulate channel inactivation kinetics as well regulating cation specificity for the channels (Wu et al., 2017; Zhao and Zlokovic, 2020). The transmembrane portion of the channel is lined by hydrophobic amino acid residues of IH, which contract in the middle of the membrane to form the neck of the channel (Zhao et al., 2018). The channel has two transmembrane gates surrounded by an OH and two adjacent IHs. Below the transmembrane channel, cations enter the cytoplasm through two intracellular channels, a 10 Å-long constriction site, and three lateral channels closed by side bolts, triggering subsequent different signaling pathways (Saotome et al., 2018; Geng et al., 2020). (Figure 1).

The transmembrane pore is followed on the inner side of the cell by two ion-permeable pathways, including a 10-Å- long vertical constriction neck along the central axis and three lateral channels extending from the central pore through three lateral openings. It is now generally accepted that the lateral channels, rather than the vertical constriction necks, may serve as the primary intracellular cation conductance pathway. The fact that the outlets of the lateral channels and the central constriction neck are surrounded by negative and positive surface electrostatic potentials, respectively, further supports the structural suitability of the lateral channels for cation permeation.

One of the key issues for piezo1 channels is how to precisely determine and regulate their precise mechanical sensitivity and cation permeation properties. Each lateral channel has a lateral “plug”. This “plug” physically controls the on/off switching of the lateral ion channel, thus affecting both the ion permeation and the mechanical sensitivity of the piezo1. The lateral cation channels, the lateral plugs, the gating element and the central constrictor neck together form the “plug and latch” mechanism of piezo1, which coordinates the switching of the lateral channels using the plugs, which are connected to the central axis to form a lever-like mechanical transduction mechanism. The three lateral ion channels are equipped with three independently positioned lateral plugs that allow the piezo1 to move asymmetrically when external mechanical forces are applied asymmetrically to the force sensing vanes, and ultimately convert the conformational changes induced by peripheral vane tension into on/off switching of the ion channels inside the cell (Geng et al., 2020).

Piezo1 is embedded in lipid bilayers and is sensitive to local and global stresses in the bilayer. It is located in the endoplasmic reticulum, the cytoplasmic compartment, and the nuclear membrane close to the nucleus (Eisenhoffer et al., 2012; Gudipaty et al., 2017). Piezo1 enables cells to sense a variety of mechanical forces, including radial pressure, membrane stretch, compression, shear stress, matrix stiffness, ultrasound, and matrix pressure (Nourse and Pathak, 2017; Ridone et al., 2019). When a mechanical stimulus impinges on the cell membrane, the stress is distributed to all components including the bilayer, cytoskeleton (CSK), and extracellular matrix (ECM), which converge on the Piezo1 channel, inducing a change from a closed to an open state of the Piezo1 channel. The Piezo1 channel opens to allow Ca^2+^, K^+^, and Na^+^ plasma flow. Under mechanical stress, Piezo1 regulates a variety of functions such as protein synthesis, secretion, migration, proliferation, and apoptosis (Hong et al., 2023). Therefore, the main factors affecting Piezo1 gating include cell membrane tension and stiffness, cytoskeletal proteins, and other channels or proteins that interact with Piezo1.

## 3 Physiological role of piezo1 in different tissues and organs and its expression in different tumors

Piezo1 channel protein is widely expressed in a variety of cells, including osteoblasts, immune cells, and cancer cells, among others. It is associated with a variety of mechanotransduction processes under different physiological and pathophysiological conditions. To emphasize the effectiveness of Piezo1 as a novel drug target for disease intervention, we summarize its physiological roles in different tissues (Table 1) and organs and its expression in different tumor tissues (Table 2).

**TABLE 1:** Comparison of piezo1 function in glioblastoma *versus* other normal tissues.

Organization	Functionality	References
Glioblastoma	• Involve in volume regulation • interferes energy supply • Cause peritumoral oedema • Regulate developmental processes • Affect the extracellular matrix • Affect microglia infiltration • Involve in abnormal angiogenesis • Enhance glioblastoma sensitivity to chemotherapeutic agents	Michelucci and Catacuzzeno (2024) Qu et al. (2020a) Chen et al. (2018) Lv et al. (2024) Knoblauch et al. (2023)
Cardiovascular	• Involve in angiogenesis and endothelial cell migration • Involve in vascular remodeling, valve morphogenesis and blood pressure regulation • Involve in lymphatic vessel development, endothelial inflammation, erythrocyte volume homeostasis	Li et al. (2014) Sun et al. (2024) Choi et al. (2022)
Bladder	• Regulate uniform and coordinated diastolic and systolic functions	Amado et al. (2024)
Kidney	• Sense intrarenal pressure changes and regulate urine osmolality	Gudipaty et al. (2017)
Bone	• Promote bone regeneration and remodeling • Regulate osteoclast differentiation and bone resorption	Guan et al. (2024)
Neural precursor cell	• Promote neuronal differentiation, neuronal glial interactions, and nanoscale neuroglial membrane development	Zhu et al. (2021)
Teeth	• Promote the reconstruction of periodontal bone tissue	Zhang et al. (2023)
Epithelial cell	• Induct cell division, regulation of cell density • Promote wound fibrosis • Prevent and minimize scar formation	Gudipaty et al. (2017)

**TABLE 2:** Expression of piezo1 in different tumor tissues and its relationship with different tumor grades and prognosis.

Tumor	Piezo1 expression	Association of higher piezo1 expression with higher tumor stage and grading	Impact of higher piezo1 expression on prognosis and survival rates	Ref.
Glioblastoma	↑	Positive correlation	↓	Qu et al. (2020b)
Oral squamous cell carcinoma	↑	-	-	Hasegawa et al. (2021)
Melanoma	↑	-	-	Patkunarajah et al. (2020)
Gastric cancer	↑	Positive correlation	↓	Chen et al. (2023)
Prostate tumor	↑	-	-	Kim et al. (2022)
Esophageal squamous cell carcinoma	↑	-	-	Gao et al. (2021)
Colon cancer	↑	Positive correlation	↓	Li et al. (2023)
Breast carcinoma	↑	-	↓	Li et al. (2024)
Ovarian cancer	↑	-		Micek et al. (2024)
Hepatocellular carcinoma	↑	Positive correlation	↓	Li et al. (2022)
Pancreatic ductal adenocarcinoma	↑	-	-	Pan et al. (2024)
Prostate cancer	↑	-	-	Kim et al. (2022)
Pancreatic cancer	↑	Positive correlation	↓	Song et al. (2022)
Non-small cell lung cancer	↓	Negative correlation	↑	Huang et al. (2019)

## 4 Relationship between Piezo1 and glioblastoma

### 4.1 Piezo1 expressed in gliomas of different grades

Neurons and glial cells in the central nervous system exhibit significant mechanosensitivity and can sense mechanical stimuli from the surrounding environment and convert them into biochemical signals to regulate their function (Cox et al., 2016; Tortorella et al., 2022). Piezo1 is expressed in neuronal cells, microglia, astrocytes, oligodendrocytes, and neural stem cells, and is activated when neuronal cells are stimulated by external tensile forces, activated when neuronal cells are stimulated by external changes in stretch force, osmotic pressure, and matrix stiffness (Hong et al., 2023), and participates in cellular physiological activities in a variety of ways, such as increasing cytoplasmic Ca^2+^ signaling or initiating Ca^2+^ inward flow and affecting the levels of downstream Ca^2+^ signaling proteins involved in neuronal function, through the c-Jun N-terminal kinase (JNK1) and mammalian target of rapamycin (mTOR) signaling pathways (Liu et al., 2021).

It was demonstrated that all histological subtypes of gliomas, except oligodendrogliomas, showed Piezo1 overexpression compared to normal brain tissue and that the expression level of Piezo1 was positively correlated with World Health Organization (WHO) grade and histopathology (Chen et al., 2018). Piezo1 expression was more significantly upregulated in high-grade gliomas (WHO grade III and IV) than in low-grade gliomas (WHO grade II). Isocitrate dehydrogenase (IDH) mutation, CpG island methylation (G-CIMP) phenotype, and 1p/19q co-deletion are important molecular biomarkers in gliomas to guide prognosis and treatment. Piezo1 expression was significantly higher in the IDH1 wild-type group of gliomas than in the IDH1 mutant group and was elevated in the 1p/19q-non-co-deletion group. Gliomas with non-G-CIMP phenotypes had higher levels of Piezo1 expression compared to G-CIMP phenotypes. Grade II oligodendrogliomas with IDH1 mutation and 1p/19q co-deletion had the lowest level of Piezo1 protein expression. IDH mutation established a glioma hypermethylation phenotype, and the deoxyribonucleic acid (DNA) structural domains spanning the Piezo1 promoter and 8000 bp upstream of the Piezo1 transcriptional start site (TSS) were also hypermethylated in IDH mutant gliomas. The methylation status of these probes was negatively correlated with Piezo1 messenger ribonucleic acid (mRNA) expression. Thus more aggressive IDH wild-type gliomas are epigenetically more inclined to upregulate Piezo1 at the transcriptional level (Zhou et al., 2020).

**FIGURE 2:**
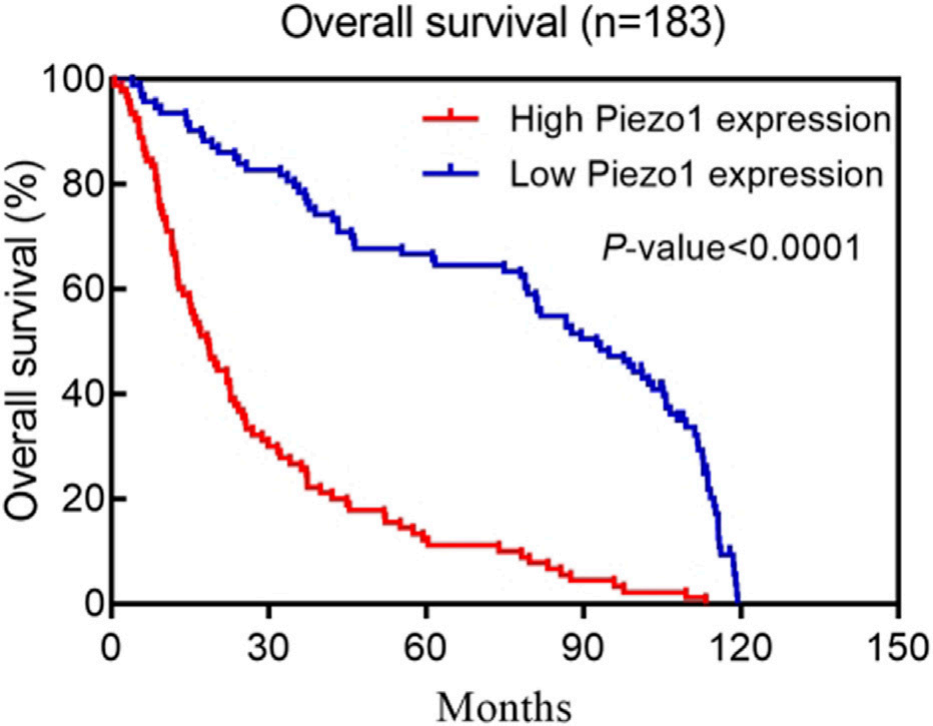
Kaplan–Meier postoperative survival curve for patterns of patients with glioma and Piezo1 expression. Adapted from (Qu et al., 2020b).

Kaplan-Meier survival analysis of multiple human glioblastoma datasets showed that patients with elevated Piezo1 expression had significantly worse overall survival (Figure 2). Multifactorial Cox regression analysis showed that Piezo1 overexpression was an independent prognostic factor for overall survival (OS) in glioblastoma patients (Qu et al., 2020b). These results suggest that Piezo1 can be used as a prognostic biomarker for glioblastoma patients in conjunction with molecular markers such as IDH wild-type, 1p/19q non-coding, and non-G-CIMP phenotypes.

Altered matrix stiffness in glioblastomas promotes elevated expression of piezo1 in glioblastomas, and Piezo1 is physically localized to the focal adhesions of glioblastoma cells, catalyzing the maturation and growth of focal adhesions through a force-dependent calcium signaling pathway. Piezo1 is one of the major ion channels conferring mechanosensitivity to GBM stem cells, and RNA of Piezo1 knockdown glioblastoma cell lines sequencing and dual-assay TCGA database analysis of Piezo1-related genes showed that Piezo1 is associated with activation of extracellular matrix (ECM), actin cytoskeleton remodeling and integrin adhesion signaling. In addition, Piezo1 expression transduces mechanical stimuli between glioblastoma cells, promoting tumorigenesis and progression (Hong et al., 2023). This finding could explain why the self-stiffening changes in glioblastomas are mainly caused by pressure gradients generated by the tumor itself rather than collagen deposition or cross-linking.

Piezo1 knockdown inhibited clonal growth of GBM cell lines. Tumor ball formation was blocked in GBM stem cells after Piezo1 knockdown. *In vitro* experiments, GBM cells were able to express Piezo1 short hairpin RNA (shRNA) in a doxycycline-dependent manner. Knockdown of Piezo1 expression by doxycycline-induced Piezo1 shRNA could be observed after treatment of glioblastoma model mice with doxycycline, which significantly suppressed GBM tumor growth and prolonged the survival time of the mice, suggesting that Piezo1 is critical for the maintenance and progression of glioblastomas once tumors form (Chen et al., 2018).

### 4.2 Involvement of piezo1 in glioblastoma cell volume regulation

Regulated volume reduction (RVD) is an evolutionarily conserved process used by animal cells to restore normal volume when osmotic shock-induced swelling occurs. RVD plays an important role in a number of physiological processes, including the prevention of necrotic cell death induced by persistent cellular swelling, and is mediated primarily by the concerted activity of ion channels that mediate the passage of CI^−^ and K^+^ ions (Miyazaki et al., 2007). Volume-regulated anion channel (VRAC), a heterodimeric protein that mediates swelling-activated CI^−^ currents (ICl), has been identified during hypotonic cell swelling as the common and major channel responsible for CI^−^ transport during RVD in almost all vertebrate cells. Tension-activated K^+^ channels, voltage-dependent K^+^ channels, and Ca^2+^-activated K^+^ (KCa) large-, medium-, and small-conductance channels (BK, IK, and SK, respectively) also play key roles in volume regulation in many cell types (Caramia et al., 2019).

In addition to CI^−^ and K^+^ channels, nonselective Ca^2+^-permeable mechanosensitive channels (MSC) are also involved in cell volume regulation. During hypotonic cell swelling, MSCs sense changes in cell membrane tension and activate as a result. Their activation then triggers intracellular Ca^2+^ signaling, which in turn regulates various effectors. (Arniges et al., 2004).

In human glioblastoma cells, Piezo1 is the main component of MSCs activated by cell swelling in glioblastoma cells, and is also primarily responsible for mechanotransduction during glioblastoma cell volume regulation. Ca^2+^ signaling can be amplified by activation of the mechanism, which is activated by the influx of MSCs activated by hypertonic cell swelling to activate IK and BK channels and VRAC, which are essential for RVD development. The cascade mechanism of Piezo1 activation from Ca^2+^ influx to IK/BK channel activation, which ultimately regulates cell volume, is an important signaling pathway for Piezo1 regulation of glioblastoma cell spreading, migration, and death (Michelucci and Catacuzzeno, 2024).

In glioblastoma tissues, the infiltration of isolated tumor cells into the peritumoral parenchyma often culminates in tumor migration and invasion. This situation greatly limits the success of surgical resection. The invasion of GBM cells into the confined space of healthy brain parenchyma requires that they be able to sense the presence of external barriers and forces from the environment and adjust their size and shape accordingly to pass through. It has been shown that glioblastoma cells use piezo1 channels to sense mechanical stimuli from the tumor microenvironment and deform the cell membrane (stretching, indentation, invagination, etc.), inducing an increase in cytoplasmic Ca^2+^ through, for example, activation of KCa channels and modulation of actin cytoskeletal polymerization, which allows for a change in the volume and shape of the cells required to invade healthy brain parenchyma.

KCa3.1 channels (also known as KCNN4, IK1 or SK4 channels) are moderately conductive calcium-activated potassium channels that regulate membrane potential and maintain calcium homeostasis. It has been shown to be involved in regulating GBM migration in response to C-X-C motif chemokine ligand (CXCL), serum and bradykinin *in vitro*. Studies have shown that the volume of invasive glioma cells is reduced by a maximum of 30%–35% compared to the volume of normal glial cells, which requires glioma cells to release all free unbound cytoplasmic water as well as K^+^ channels. Water permeably follows the release of ions through ion channels and cotransporter proteins, with a consequent change in cell volume. Inhibition of KCa3.1 channels retards cell invasion and migration (Watkins and Sontheimer, 2011).

Volume changes are critical in cell death, such as in apoptotic cell death, which is preceded by a contraction of cell volume called apoptotic volume decrease (AVD). In GBM cells, KCa channels are directly involved in AVD induced by the addition of astrocystin or TNF-α-related apoptosis-inducing ligand (TRAIL), which activate intrinsic or extrinsic pathways of apoptosis, respectively.

### 4.3 Piezo1 interferes with the energy supply in glioblastomas

Ca^2+^ regulates glioblastoma maintenance, proliferation, migration, and cell cycle (Leclerc et al., 2016; Satir and Christensen, 2007). Most cancer cells obtain higher glucose utilization under aerobic conditions by producing ATP using glycolysis, a phenomenon known as the Warburg effect (Vander Heiden et al., 2009). When intracellular glucose is deficient, free fatty acid oxidation replaces glucose for cellular energy and maintains intracellular reactive oxygen species (ROS) levels and calcium homeostasis, Ca^2+^ is closely related to lipid metabolism (Li et al., 2018; Wires et al., 2017).

Sonodynamic therapy (SDT) is a non-invasive technique based on the combination of acoustic sensitizers and acoustic activation that disrupts the mitochondrial respiratory chain, leading to an increase in intracellular ROS levels and calcium overload, as well as inhibition of proliferation, invasion, and promotion of apoptosis in the biologically more aggressive grade IV glioblastomas (Chen et al., 2022). On the one hand, the mechanical force introduced by ultrasound activated the opening of the transient calcium channel Piezo1 on the membrane, maintained the open state of the Piezo1 channel by increasing the level of intracellular oxidative stress, prolonged the opening time of Piezo1, and increased the Ca^2+^ inward flow, which caused the cell to swell and activated the calcium overload pathway of the death mechanism. On the other hand, ROS generated by ultrasound-activated intracellular ultrasound sensitizers prolonged the opening time of Piezo1 and promoted Ca^2+^ entry into the cell to bind to lipid droplets to form complexes, thereby affecting lipid metabolism and preventing it from providing energy to glioblastoma cells, leading to cell death (Chen et al., 2022).

ROS generated by SDT activates the mitochondrial apoptotic pathway (Chen et al., 2017), leading to mitochondrial dysfunction and DNA damage, which sustains mitochondrial calcium uniporter (MCU) channel activity and induces Ca^2+^ overload in the mitochondria, further aggravating the mitochondrial dysfunction, generating more ROS, and forming a vicious cycle that ends in death.

### 4.4 Peritumoral oedema in glioblastoma caused by Piezo1

Peritumoral oedema is an independent risk factor for poor prognosis and recurrence of glioblastoma (Schoenegger et al., 2009). Peritumoral oedema can exacerbate neurological signs and clinical symptoms in glioblastoma patients, such as seizures, neurological deficits, and increased intracranial pressure, and can even lead to brain herniation. In addition, peritumoral oedema can affect tumor exposure during surgery and make surgical resection more difficult (Wang et al., 2011; Hou et al., 2013). A recent study reported a linear correlation between the expression level of Piezo1 and the severity of peritumoral oedema, with preoperative magnetic resonance imaging (MRI) and corresponding Piezo1 immunohistochemical staining results in patients with different degrees of oedema. The higher the degree of oedema, the higher the Piezo1 expression (Qu et al., 2020a).

Peritumoral brain oedema (PTBE) includes vasogenic and cytotoxic oedema (Qu et al., 2020a; Liang et al., 2007). Vasogenic oedema is usually due to the degradation of tight junctions between endothelial cells (e.g., E-cadherin, N-cadherin, and b-catenin), which increases vascular permeability. Intravascular fluid then leaks out into the tissue space, further causing tissue oedema. The Piezo1 protein acts as a calcium channel to promote calcium ion influx into vascular endothelial cells, which in turn activates calcium-dependent calpain. Calpain further promotes the degradation of tight junctions between vascular endothelial cells and increases vascular permeability (Friedrich et al., 2019). High vascular permeability allows the extravasation of protein-rich fluid, leading to cerebral oedema. Extracellular cations enter neurons and glial cells through cation channels and accumulate intracellularly, while cation in-flow drives anion inflow, leading to increased intracellular osmotic pressure and entry of extracellular interstitial water molecules into the cells, resulting in cellular swelling and the formation of cytotoxic oedema occurs. Thus, inhibition of Piezo1 reduces clinical symptoms in glioblastoma patients and may be a therapeutic target for diseases involving blood-brain barrier collapse (Qu et al., 2020a).

### 4.5 Pathways regulated by piezo1 during glioblastoma development

#### 4.5.1 JNK1/mTOR

The ROS-c-JunN-terminalkinase 1 (JNK1) and AKT-mammalian target of rapamycin (mTOR) signaling pathways are the main molecular mechanisms involved in glioblastoma cell survival, proliferation, motility, and differentiation, and the activation and activity of this signaling pathway is regulated by intracellular Ca^2+^ signaling (Mohamed et al., 2022; Park et al., 2008). Activation of cell cycle proteins D1 and cyclin-dependent kinases 4 (CDK4) and assembly of the cell cycle protein D1-CDK4 complex are essential for promoting the cell cycle transition from G1 to S phase. Highly expressed and aberrantly activated piezo1 in glioblastoma induces calcium inward flow. Large amounts of intracellular Ca^2+^ promote phosphorylation of Akt and mTOR, followed by activation of the cell cycle protein D1/CDK4 complex, which promotes cell survival, cell cycle progression, cell proliferation, and migration. In addition, since Akt stabilizes the mature cell cycle protein D1, piezo1-induced activation of Akt can promote the transition of tumor cells from G1 to S phase by activating the cell cycle protein D1. knockdown of Piezo1 may inactivate Akt, inhibit the activation of the cell cycle protein D1, and cause the cells to stagnate in the G1 phase (Han et al., 2019). Meanwhile, studies have shown that inhibition of Piezo1 further increases the expression of P-JNK1 (T183/Y185), promotes the expression of pro-apoptotic proteins such as c-Jun N-terminal kinase (cJUN), Bcl2-associated X (BAX), etc., inhibits tumor cell proliferation, and induces apoptosis of glioma cells (Liu et al., 2019).

Celastrol is a major active natural product extracted from the root bark of the traditional Chinese medicine Rehmannia glutinosa, which possesses a wide range of biological properties such as anti-tumor, immunosuppression, and weight loss (Liu et al., 2015; Hu et al., 2017b). Previous studies have shown that celastrol inhibits the growth of rat glioblastoma cells and human glioblastoma cells by inhibiting VEGFR expression, inducing apoptosis and cell cycle arrest (Huang et al., 2008; Ge et al., 2010). Recent studies have shown that celastrol triggers apoptosis and autophagy by activating the ROS/JNK signaling pathway and blocking the AKT/mTOR signaling pathway, inducing G2/M phase block, and in a dose-dependent manner. *In vivo* experiments showed that celastrol increased the levels of cleaved Caspase-3, LC3B, and p-JNK, decreased the expression of p-AKT and mTOR, and inhibited the growth of tumors in a mouse glioblastoma *in situ* transplantation model. Naïve T cells are relatively quiescent until exposed to antigenic stimulation, they are metabolically quiescent and have low nutrient uptake, glycolytic rate and biosynthesis. Calcium store-operated calcium entry (SOCE) directs the proliferation of naïve T cells and adaptive immune responses by activating the nuclear factor of activated T cells (NFA T) and the PI3K-AKT-mTOR pathway regulating the expression of glucose transporter proteins, glycolytic enzymes, and metabolic regulators (Vaeth et al., 2017). It was shown that both channels are regulated by intracellular Ca^2+^ signaling and were shown to be involved in the downstream mechanisms of Piezo1 (Liu et al., 2019).

#### 4.5.2 FAKs

Adhesion zones are hubs of cytoskeletal structures connecting the extracellular matrix to the intracellular skeleton, and they detect and transmit external mechanical forces. Focal adhesion kinase (FAK), a member of the adhesion zone proteins, is a key regulator of adhesion plaque dynamics and cytoskeletal remodeling, which exhibits high expression in a wide range of tumors and correlates with poor prognosis. FAK activation promotes tumor growth, invasion, metastasis, and angiogenesis through kinase-dependent (such as PI3K/AKT signaling pathway, P53 signaling pathway, RAS/RAF/MEK/ERK signaling pathway, YAP signaling pathway) and kinase-independent pathways (e.g., inhibition of T-cells, B-cells, and dendritic cells in the immune microenvironment. Promote myeloid-derived suppressor cells (MDSC), tumor-associated macrophages (TAM), cancer-associated fibroblasts (CAF), and so on.) (Hu et al., 2024; Kim et al., 2011). Piezo1 interacts with FAK and signals to transcriptional co-activators through the pdz-binding motif (TAZ), leading to chromatin remodeling and changes in transcript levels (Hu et al., 2017a). Tumor tissue sclerosis provides a mechanistic microenvironment for the activation of Piezo1, which promotes adhesion plaque assembly and activates the β1-integrin-FAK signaling pathway, regulates cell proliferation and participates in extracellular matrix remodeling, which further regulates tissue stiffness and the stiffer environment further enhances Piezo1 expression, which increases mechanosensory and mechanotransduction capacities of tumor cells (Chen et al., 2018). Tumor cells thus form a feedback loop between Piezo1-dependent mechanosensing and abnormal tissue mechanics that interact and exacerbate disease. Integrin inhibitors (such as cilengitide or etanercept) have been tested in clinical trials. A randomized phase II trial demonstrated that cilengitide monotherapy was well tolerated and had modest anti-tumor activity against recurrent glioblastoma (Chen et al., 2018). However, a phase III trial demonstrated that cilengitide in combination with standard temozolomide radiotherapy did not have better efficacy than conventional treatment in glioblastoma, particularly in tumors with methylated O6 -methylguanine-DNA methyltransferase (MGMT) promoters, and the use of cilengitide as a subunit antagonist of the integrins αvβ3 and αvβ5 is currently still controversial (Desgrosellier and Cheresh, 2010).

#### 4.5.3 YAP

**FIGURE 3:**
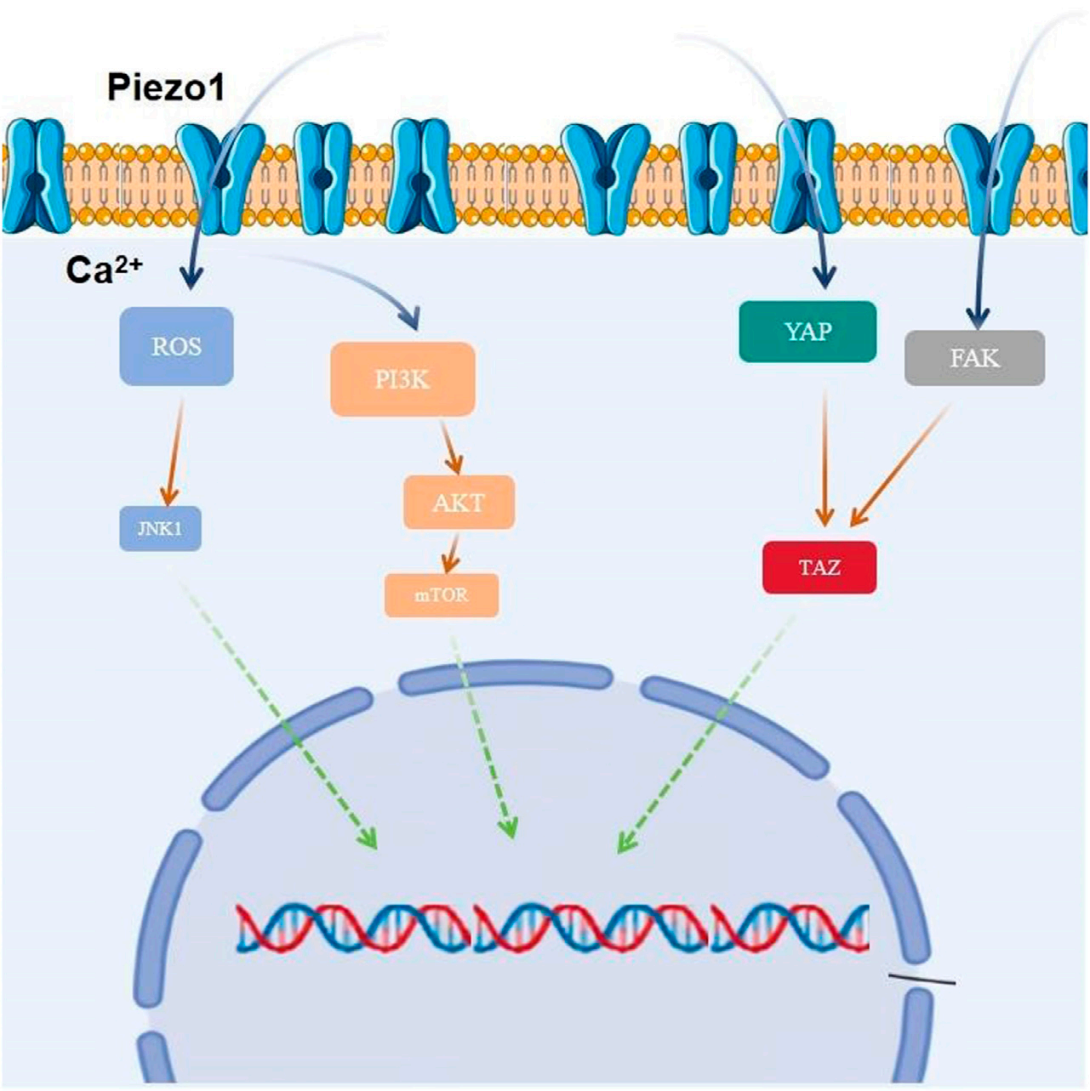
Piezo1 is involved in glioblastoma proliferation by regulating multiple signaling pathways.

Yes-associated protein (YAP), an important co-transcription factor of the Hippo signaling pathway, plays an important role in mechanistic effects and the malignant proliferation and invasion of tumor cells. YAP/transcriptional coactivator with PDZ-binding motif (TAZ) is an effector of mechanistic signaling stimuli of matrix stiffness, stretch, and cell density. Mechanically relevant events such as altering mechanical stimuli inside and outside the cell, inoculating cells with different matrix stiffnesses, exposing cells to periodic stretching motions, and adjusting the degree of cell adhesion to the extracellular matrix can cause activation of YAP/TAZ in the nucleus (Zanconato et al., 2016). Abnormalities in YAP function have been closely associated with glioblastomagenesis, cellular proliferation, maintenance of tumor cell stemness, and invasive metastasis. Matrix stiffness can promote glioblastoma cell proliferation, EMT, and angiogenic mimicry formation through YAP, etc (Castellan et al., 2021). Piezo1 acts as a mechanosignal receptor, transmits extracellular matrix stiffness signals, and promotes glioblastoma cell proliferation, invasion, and epithelial-mesenchymal transition by regulating the nuclear expression and activity of YAP and subsequent TAZ activation. The study demonstrated that the piezo1 agonist Yoda1 significantly increased intracellular Ca^2+^ concentration in the cytoplasm of valvular mesenchymal stromal cells, whereas piezo1 knockdown reduced Ca^2+^ influx. Simultaneous immunofluorescence determination of YAP subcellular localization demonstrated that Yoda1 triggered YAP nuclear translocation, whereas piezo1 knockdown prevented cytoplasmic YAP from migrating to the nucleus. Using the calcium chelator1,2-bis (2-aminophenoxy)ethane-n,n,n0,n0-tetraacetic Acid Tetrakis (acetoxymethyl Ester) (BAPTA-AM) to reduce intracellular Ca^2+^ concentration, it was observed that BAPTA-AM eliminated Yoda1-induced intracellular Ca^2+^ accumulation and that BAPTA-AM inhibited Yoda1-induced YAP nuclear translocation, resulting in YAP being mostly localized in the cytoplasm. These results suggest that intracellular calcium accumulation is responsible for the YAP nuclear translocation induced by piezo1 (Zhong et al., 2023). Meanwhile, it was found that YAP was located downstream of β1-integrin-RhoA, which was an important effector of cellular response to matrix signals and mechanical stimuli (Zoi et al., 2022). Downregulation of the expression of Piezo1 had a significant effect on the β1-integrin/FAK pathway, and also significantly affected the nuclear localization and activity of YAP, suggesting that Piezo1 promotes the progression of glioblastoma invasion through the β1-integrin/FAK/YAP axis. invasion progression. It was shown that knockdown of the Piezo1 gene in glioblastomas using interfering shRNA lentiviral vectors resulted in a significant increase in the expression of the cellular epithelial marker E-cadherin, and a significant decrease in the expression of the mesenchymal markers waveform protein, SNAIL, and Slug. This suggests that the expression of Piezo1 regulates EMT, and maintains mesenchymal characteristics of glioblastoma cells. characteristics of glioblastoma cells (Figure 3).

### 4.6 Relationship between Piezo1 and glioblastoma exosomes

Exosomes are small extracellular vesicles (EVs) with a diameter of 40–160 nm (average ∼100 nm) that mediate intercellular communication (Kalluri and LeBleu, 2020). Exosomes contain components such as proteins, lipids, DNA, mRNA, and non-coding RNAs, which play important roles in tumor angiogenesis, invasion, metastasis, and drug resistance. Cyclic RNAs are a novel class of RNA transcripts produced by head-to-tail splicing of exons, which are produced and structured differently from mRNAs and therefore have unique cellular functions and potential biomedical applications (Memczak et al., 2013). Studies have shown that circular RNAs play an important regulatory role in glioblastoma. circZNF800 is a recently identified circular RNA that is highly expressed in glioblastoma stem cells. Studies have shown that glioblastoma stem cells secrete exosomes to enhance GBM cell proliferation and migration and inhibit apoptosis in glioblastoma. highly expressed circZNF800 regulates the expression of tumor-associated genes through the circRNA/PI3K/AKT axis and promotes glioblastoma progression (Zhang et al., 2024).

Micro RNAs (miRNAs) are small, highly conserved, small non-coding RNA molecules that control gene expression at the post-transcriptional level. It inhibits translation or promotes degradation of mRNAs by binding to complementary binding sites within the 3‘ untranslated region (3’UTR) of target messenger RNAs (mRNAs)(Bartel, 2004). miRNA-139 is located at 11q13.4 and exhibits anti-tumor and anti-metastatic activities in humans. It was shown that miR-139-5p was significantly reduced and negatively correlated with circZNF800 expression in GBM tissues compared with normal brain tissues. miR-139-5p inhibitor significantly promoted the expression of p-AKT protein, which further promoted the invasion of U251 and U87 cells and inhibited apoptosis of GBM cells, whereas the downregulation of circZNF800 eliminated this effect. Piezo1, a direct target of miR - 1395p, was regulated by circZNF800, and correlation analysis showed a positive correlation between the expression levels of circZNF800 and Piezo1 mRNA. In GBM cells, miR-139-5p significantly reduced the expression of Piezo1 mRNA. Whereas overexpression of circZNF800 significantly increased Piezo1 mRNA expression. CircZNF800 activated the Piezo1/AKT signaling pathway through phagocytosis of miR-139-5p and regulated proliferation, migration, and apoptosis of GBM cells (Zhang et al., 2024).

### 4.7 Piezo1 affects the extracellular matrix of glioblastomas

The extracellular matrix is the general scaffold that maintains the homeostasis of tissues and organs *in vivo*. It is also a key component of the cancer microenvironment that supports tumorigenesis. During tumor development and progression, a complex ECM network is established by fibrous or nonfibrous collagen, elastin, proteoglycans, glycoproteins, laminin, fibronectin, and other matrix proteins. The extracellular matrix not only provides nests for cancer cells and stromal cells, but also serves as a reservoir for growth factors and cytokines. In addition, the ECM interacts with stromal, parenchymal, precancerous or cancer cells to initiate different cell signaling cascades that stimulate cellular differentiation metastasis, autophagy, epithelial-mesenchymal transition (EMT), cell migration, invasion and metabolic reprogramming to promote cancer cell proliferation, migration, invasion, angiogenesis, and immune evasion. mechanical stimuli induced by both the ECM *per se* and by ECM sclerosis can activate cell membrane receptors and mechanosensors such as integrins, piezo1 and transient receptor potential ion channel subfamily V 4 (TRPV4), thereby modulating the malignant phenotype of tumor and stromal cells. In the glioblastoma tumor microenvironment, Piezo1 plays a particularly strong role in the ECM signaling pathway.

As a therapeutic target for glioblastomas, Piezo1 senses microenvironmental stiffness and converts mechanical stimuli into electrical and chemical signals that promote calcium influx. Elevated intracellular Ca^2+^ concentration catalyzes the assembly and maturation of focal adhesions and directly activates the integrin-focal adhesion pathway, which stimulates cell proliferation and regulates extracellular matrix remodeling, further increasing glioblastoma tissue stiffness in positive feedback. Enhanced stiffness between glioblastoma cells and the ECM microenvironment in turn regulates the activation of Piezo1.

Tumor cells and stromal cells can respond to mechanical signals induced by matrix sclerosis. ECM sclerosis typically induces mechanical perturbation of the lipid bilayer and activation of transient receptor potential (TRP) family channels and piezo1 channels, evolutionarily conserved ion channels that link ECM sclerosis-associated mechanical forces to cellular signaling pathways, particularly Ca^2+^ signaling in tumor and stromal cells. The cell surface receptor integrin is a mechanosignal transducer that can be activated by Piezo1 to promote cancer stem cells and drug resistance. Physical interactions between the extracellular structural domains of integrins and ECM proteins induce the assembly of cytoplasmic complexes consisting of scaffolding proteins (neuregulin, talin, stumpins, etc.), adhesion plaque kinase (FAK), Src, and PI3K/Akt, which orchestrate adhesion plaque and cytoskeletal assembly with stromal mechanical cues. The Rap1 GTPase also responds to the cytoplasmic signaling of integrins by stabilizing integrins and recruiting neuregulin to the adhesion plaque. Rap1 GTPase also responds to matrix sclerosis by stabilizing integrins and recruiting connexins to adhesion patches. In addition, Rho-associated coiled-coil kinases (ROCK) activation may be induced by ECM sclerosis, which then promotes integrin signaling, mitogen-activated protein kinase (MAPK) activation, and SNAIL stabilization. Integrins, integrin-linked kinase (ILK), SNAIL, and Src upregulate YAP expression and activation, thereby upregulating piezo1 expression.

Piezo1 senses the stiffness of the microenvironment and converts mechanical stimuli into electronic and chemical signals that promote calcium influx. Elevated intracellular Ca^2+^ concentrations catalyze the assembly and maturation of focal adhesions, and piezo1 activates the integrin-FAK pathway when the extracellular structural domains of integrins interact with ECM proteins such as collagen, laminin, and tendon proteins, whose cytoplasmic structural domains are complexed with scaffolding proteins, including ankyrin and stumpyrin, as well as kinases, such as adhesion patch kinase (FAK) and Src. Through integrin-taIin, talin-FAK interactions, multiple FAK molecules accumulate at the focal adhesion site, resulting in the activation of FAK by phosphorylation and its association with other signaling molecules. Upon activation of FAK, its phosphorylated Tyr397 binds to the Src family of kinases, causing the PAK/Src complex by forming the FAK/Src complex. Src complex and causes phosphorylation of Paxillin and clustered regularly interspaced shortpalindromic repeats (CRISPR)-associated systems (Cas), which activate mitogen-activated protein kinase (MAPK), a kinase downstream of the rat sarcoma (Ras) pathway, via the junction proteins cysteine-rich receptor-like protein kinases (Crk) and growth factor receptor-bound protein 2 (Grb2) to control lesion adhesion and cytoskeleton assembly, regulate extracellular matrix remodeling, and increase the stiffness of glioblastoma tissues. and activate integrin-dependent intracellular kinase signaling to regulate cell adhesion, motility, proliferation, survival and differentiation (Chen et al., 2018).

It was shown that GBM stem cells were cultured in polyacrylamide hydrogels at different stiffness levels (100-5,000 Pa) in the normal human brain, low-grade glioblastomas, and high-grade glioblastomas. The percentage of proliferatively active GBM stem cells and the total number of cells increased with increasing stiffness, while Piezo1 knockdown eliminated this stiffness-dependent tumor cell growth (Chen et al., 2018).

**FIGURE 4:**
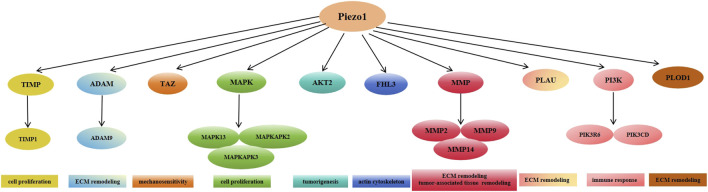
Expression of Piezo1 positively correlates with multiple genes mediating extracellular matrix remodeling in glioblastoma.

Furthermore, many signaling pathways such as matrix metalloproteinase (MMP) family, tissue inhibitor of metalloproteinases (TIMP) family, mitogen-activated protein kinase (MAPK) family, phosphoinositide 3 kinase (PI3K) family, and others are positively correlated with high expression of Piezo1 during the pathology of glioblastoma development. Various pathways such as the mitotic cell cycle, focal adhesion, ECM, and tumor cell response to mechanical stimuli were altered after the knockdown of Piezo1. In the CGGA and TCGA databases, Piezo1 expression was positively correlated with the genes TIMP1, MMP2, MMP9, MAPK13, MAPKAPK2, PIK3R6, PIK3CD, PLOD1, and AKT2, and was also positively correlated with the genes TIMP1, MMP2, MMP9, MAPK13, MAPKAPK2, PIK3R6, PIK3CD, PLOD1, and AKT2, as opposed to low-grade glioblastomas (WHO classification II and WHO classification III) in glioblastomas (WHO grade IV) had significantly higher expression of all ECM-related genes positively associated with Piezo1. Among these genes, TIMP1, MAPK13, MAPKAPK2, and MAPKAPK3 regulate cell proliferation, PLOD1, MMP14, ADAM9, and PLAU control ECM remodeling, MMP2, and MMP9 control tumor-associated tissue remodeling; PIK3CD is involved in immune response, AKT2 regulates tumorigenesis, FHL3 controls actin cytoskeleton, and TAZ regulates the mechanosensitive HIPPO signaling pathway (Figure 4). Taken together, these data suggest that Piezo1 not only delivers mechanical inputs to promote glioblastoma growth but also positively regulates the ECM and other mechanotransduction mechanisms in tumors (Zhou et al., 2020).

### 4.8 Piezo1 contributes to the immunosuppressive tumor microenvironment in glioblastoma

In healthy brain tissue, glioblastoma-associated microglia (GAM) are highly branched, and branched microglia usually exhibit properties of M1-type macrophages that secrete pro-inflammatory factors to destroy pathogens. When inflammation, infection, trauma, or other neurologic diseases occur in the brain, microglia transform to the M2-type macrophage form. The transformed microglia have enlarged cell bodies, shortened protrusions, amoeboid morphology and activated phagocytosis. (Gieryng et al., 2017). GAM is the predominant inflammatory cell in the tumor micro-environment (TME), which not only exerts an immunosuppressive function and induces immune tolerance, but also promotes tumor growth and metastasis (Lin et al., 2024). Additionally, studies have shown that the immunosuppressive phenotype of GAMs correlates with GBM grade, with higher grades showing greater prevalence of tolerant or protumor GAMs (De et al., 2016). Neovascularisation in malignant tumors is one of the features that distinguish them from benign tumors, and the results of both clinical and experimental studies support the production of angiogenic factors [e.g., VEGF] by GAM to stimulate angiogenesis in glioblastomas. Studies have shown that IL-6 released from GAM keeps the blood-brain barrier in glioblastoma patients highly permeable by activating the Janus kinase 2/signal transducer and activator of transcription 3 (JAK/STAT3) pathway in endothelial cells and down-regulating the levels of intercellular junction proteins, which is responsible for the formation of vasogenic brain oedema (Couto et al., 2019). GAM-released IL-1β can also be used to stimulate the formation of vasculogenic brain oedema in glioblastoma cells by promoting glycerol-3-phosphate dehydrogenase 2 (GPD2) phosphorylation and glycolysis, thereby accelerating tumor proliferation and growth (Lu et al., 2020). In addition, microglia induce platelet-derived growth factor receptor (PDGFR) expression in mouse glioblastoma models and tumor cells from human patients, and the expression of this receptor stimulates glioblastoma cell migration, thereby accelerating tumor progression (Sherafat et al., 2021; Wallmann et al., 2018). Piezo1-mediated increases in cytoplasmic Ca^2+^ are involved in the inhibition of microglia proliferation. Piezo1 opening promotes extracellular Ca^2+^ endocytosis and releases Ca^2+^ stored in the endoplasmic reticulum into the cytoplasm, creating a cytoplasmic Ca^2+^ peak that interferes with the homeostasis of endoplasmic reticulum-mitochondrial Ca^2+^ communication and induces microglial dysfunction. Piezo1 is recruited to the endoplasmic reticulum via the R-Ras (which is most likely in an activated state), and then stimulates calcineurin signaling to maintain integrin activation, thereby enhancing cell adhesion. *In vitro*, microglia cultured on hardness-gradient hydrogels migrate to harder regions and are more responsive in these regions, suggesting that microglia may migrate towards and function in harder tumor tissue *in vivo*. Inhibition of Piezo1 expression modulates the affinity for integrins within microglia, thereby increasing the rate of cell migration (Hu et al., 2023). Bone marrow-derived macrophages (BMDM) migrate to the tumor site and accumulate in the central region of the GBM, exerting a predominantly immunosuppressive effect, whereas resident microglia have little or no immunosuppressive function. In grade II and III glioblastomas, microglia accounted for the majority of macrophages and lacked significant immunosuppressive activity (Miller and Hjelmeland, 2023). Activation of Piezo1 channel proteins by mechanical stimulation regulates macrophage functional differentiation, modulates dendritic cells (DC) responses triggers inflammation, and participates in T-cell activation, among many other ways, in immune cell infiltration in the tumor microenvironment (Pinton et al., 2019).

Mechanical signaling has been identified as a potential trigger for inflammatory activation in microglia. Piezo1 has the ability to regulate microglial migration patterns and immune responses, and has been suggested to act as a mechanosensor for microglial cell lines. Piezo1 regulates the pro-inflammatory response of microglial cells upon activation by lipopolysaccharide (LPS), a commonly used surrogate for bacterial infections, inducing a transient changes. *In vitro* and *in vivo*, deletion of the Piezo1 gene in microglia reduced LPS-induced proinflammatory cytokine production, whereas Yoda1 increased LPS-stimulated proinflammatory cytokine production in microglia. Similar to microglia, macrophages lacking the Piezo1 gene exhibited reduced inflammation under both resting and endotoxin-stimulated conditions, accompanied by decreased proinflammatory cytokine production. These alterations may be related to Piezo1-mediated Ca2+influx and crosstalk between cytoskeletal and integrin proteins and piezo1 (Hu et al., 2023). While at the same time, some studies have shown that LPS can lead to microglia activation via Toll-like receptor 4 (TLR4), leading to activation of NF-κB signaling via the extracellular signal-regulated kinase 1/2 (ERK1/2) and p38 mitogen-activated protein kinase (MAPK) pathways, which ultimately drives TNF-α and IL-6 expression and production. However Yoda1 inhibits LPS-induced NF-κB activation by activating piezo1-Ca2+ signaling. These results suggest that activation of piezo1 channels reduces LPS-induced microglia activation and proinflammatory cytokine production, and that NF-κB signaling may act as a major mediator of (Malko et al., 2023).

In addition to this, ROS generated by SDT stimulation interferes with mitochondrial oxidative respiration, allowing more Ca2+ to enter the mitochondria, leading to mitochondrial calcium overload and dysfunction. MCU inhibition due to mitochondrial dysfunction prevents mitochondrial calcium uptake and promotes M1 polarization (Tedesco et al., 2019). Early intervention of Ca2+ channels in combination with SDT increases the proportion of M1-type macrophages with pro-inflammatory and anti-tumor effects.

Piezo1, integrins, and YAP are also strongly associated with cancer immunity. High collagen density and piezo1 activation promote macrophage polarization and enhance their immunosuppressive phenotype, leading to a decrease in cytotoxic T cell numbers and proliferation and inducing the expansion of immunosuppressive myeloid cells. Studies have shown that deletion of piezo1 in T cells does not affect effector T cell function but increases immunosuppressive myeloid cell, suggesting that activation of piezo1 in T cells may enhance the immune response in autoimmune diseases. In addition, increased matrix stiffness impedes the ability of dendritic cells to elicit an immune response *in vitro* and inhibits dendritic cell migration, which is essential for activating T cells and eliciting an immune response. necessary for T cells and eliciting an immune response (Jiang et al., 2022).

### 4.9 Involvement of Piezo1 in abnormal angiogenesis in glioblastoma

Increased intracellular Ca^2+^ concentration plays a crucial role in angiogenesis and arterial remodeling (Moccia et al., 2014; Moccia et al., 2012). Growth factors and cytokines, such as vascular endothelial growth factor (VEGF), epidermal growth factor (EGF), basic fibroblast growth factor (bFGF), insulin-like growth factor-1 (IGF-1), angiopoietin, and stromal-derived factor-1α (SDF-1α), trigger potent Ca^2+^ signaling in vascular endothelial cells (Potenza et al., 2014; Moccia et al., 2003; Noren et al., 2016; Munaron and Fiorio Pla, 2000) recruiting large numbers of downstream Ca^2+^-dependent pro-angiogenic transcription factors. These transcription factors include but are not limited to the nuclear factor of activated t-cells (NFAT), nuclear factor κ b (NF-κB) and cyclic adenosine monophosphate (cAMP) -response element binding protein (CREB) (Noren et al., 2016; Zhu et al., 2008; Chen et al., 2016), myosin light chain kinase (MLCK) and myosin 2 (Tsai et al., 2014), endothelial-type nitric oxide synthase (eNOS)(Berra-Romani et al., 2013; Charoensin et al., 2017), extracellular signal-regulated kinase 12 (ERK 1/2) and AKT (Lyubchenko et al., 2011). Subsequent studies have demonstrated that endothelial Ca^2+^ signaling may also drive tumor angiogenesis, growth, and metastasis. Piezo1 regulates glioblastoma angiogenesis by engaging the Ca^2+^-dependent proteins eNOS and calpain, which promote the migration, alignment, and rearrangement of vascular endothelial cells in the direction of blood flow (Zhou et al., 2020; Barnes et al., 2017).

Piezo1 is highly expressed and co-localized in extensively angiogenic GBM endothelial cells and promotes aberrant angiogenesis in glioblastoma through the HIF-1α/VEGF pathway. Myosin 1b promotes angiogenesis through the VEGF/myc/myosin 1b/piezo1 axis. myosin 1b can block the degradation of hypoxia-inducible factor 1α (HIF-1α) (a marker of hypoxia) by inhibiting autophagosome-lysosome fusion to promote the expression of VEGF. myc is a transcription factor of myosin 1b, which is induced by VEGFA. Myc is a transcription factor of myosin 1b and plays a key role in VEGFA-induced myosin 1b expression. myosin 1b regulates the expression of Piezo1 and Ca^2+^ influx, and Piezo1 knockdown reverses VEGF-induced intracellular Ca^2+^ influx. Ca^2+^ influx induced by vascular endothelial growth factor (Lv et al., 2024).

## 5 Targeting Piezo1 is a new direction for treating GBM

Given that Piezo1 is expressed in a wide range of cells and is involved in a variety of physiopathological processes, targeting this mechanosensitive protein would constitute a novel strategy for treating certain pathological conditions. Many studies have investigated the ionotropic and mechanogating mechanisms of Piezo1 channels.

### 5.1 Non-competitive inhibitors of Piezo1

The amphiphilic peptide toxin GsMTx4 interferes with Piezo1 by modulating local membrane tension and does not act directly on Piezo1 itself (Suchyna, 2017). In unstressed membranes, GsMTx4 occupies a small region of the bilayer surface, stabilized by the positive charge of lysine residues. When pressure is applied to force the lipid to stretch, GsMTx4 sinks deeper into the membrane, forming a ‘regional reservoir’ to ‘clamp’ the tension applied to the outer layer. As a result, the efficiency of stimulus transport to the Piezo1 channel is disrupted, thereby inhibiting the Ca^2+^-mediated AKT/mTOR pathway, which plays a crucial role in tumor development (Gnanasambandam et al., 2017). Due to its specificity as a cationic mechanosensitive channel inhibitor, GsMTx4 has been widely used to study the physiological and pathological roles of Piezo1 channels. An increasing number of studies have begun to explore the potential of GsMTx4 as a new strategy for glioblastoma treatment (Chen et al., 2022).

Tetradecylamine-1-κ5n,2-κ5n,3-κ4n-di-μ-oxohexachlorotrisruthenium, also known as ruthenium red (RR), is a blocker of Piezo1-induced mechanosensitive currents, and has been widely used in biological studies as a non-selective inhibitor of calcium channels (Tapia and Velasco, 1997). RR inhibits the inward, but not the outward, mPiezo1 MA currents, suggesting the presence of a pore-blocking mechanism (Coste et al., 2012).

Gadolinium (Gd^3+^), a trivalent lanthanide, can inhibit Piezo1 by binding to intramembrane anionic phospholipids interfering with neighboring membrane lipids and does not block Piezo1 by directly blocking the channel pore (Ermakov Yu et al., 1998). Gd^3+^ efficiently binds to intramembrane anionic phospholipids such as phosphatidylserine, with a consequent increase in the membrane side pressure, which further stabilizes the Piezo1 channel towards the closed state (Ermakov et al., 2010).

Although ruthenium red and gadolinium exhibit inhibitory properties of piezo1, both are prone to off-targeting when acting as piezo1 inhibitors because they are non-specific blockers of many stretch-activated cation channels.

Amyloid (aβ) is a group of peptides that play a key role in the pathogenesis of Alzheimer’s disease. Similar to GsMTx4, the amphiphilic nature of Aβ peptides suggests that they can modulate membrane structure. Aβ peptides can affect cellular mechanotransduction by inhibiting Piezo1 activity. Accumulation of Aβ peptides is closely associated with upregulation of Piezo1 in reactive astrocytes (Velasco-Estevez et al., 2018) and microglial cells (Jäntti et al., 2022).

Saturated and polyunsaturated fatty acids Dietary fatty acids are important components of cell membranes and have a significant impact on their mechanical properties (e.g., bending stiffness and viscosity). Saturated margaric acid (n-heptadecanoic acid) stabilized the closed state of Piezo1 channels by increasing membrane order and binding stiffness in a concentration-dependent manner, inhibiting of Piezo1 channels (Romero et al., 2019). Several polyunsaturated fatty acids (PUFAs) are also negative modulators of Piezo1, e.g., arachidonic acid (AA) or eicosapentaenoic acid, either by mediating a metastable coupling between the inner pore helix (IH) and c-terminal extracellular structural domains (CED) or by disrupting the regulation of Piezo1 by other membrane proteins (Thien et al., 2024).

### 5.2 Competitive inhibitors of Piezo1

Tubeimoside I is a triterpenoid saponin extracted from the Chinese herb Bolbostemmatis Rhizoma, whose sugar chain is linked by 3-hydroxy-3-methylglutaric acid, forming a unique macrocyclic structure. Tubeimoside I had a significant inhibitory effect on Ca^2+^ responses in 293T cells overexpressing piezo1 by Yoda1. Tubeimoside I was also relatively selective for Pio 1 because it did not block Ca^2+^ currents induced by other mechanical actions. A negative correlation was shown between the concentration of Yoda1 and the inhibitory response of Tubeimoside I, and the two compounds may reversibly compete with each other for piezo1 in the Yoda1 binding site (Liu et al., 2020).

It was shown that TBMS1 inhibited viability and AKT phosphorylation in U251 cells. PI3K/AKT inhibitor lY294002 showed the same effect. Furthermore, the combination of TBMS1 and lY294002 enhanced these inhibitory effects (Cao et al., 2020).

The cell cycle control system is based on two major protein families, cell cycle proteins and cdKs. Normally, the expression levels of cell cycle proteins are dynamic, whereas cdKs act as catalytic subunits that stabilize the expression levels of cell cycle proteins. During the transition from G2 to M phase, cyclin B1 activates CDK1, leading to an increase in activated CDK1 (Arellano and Moreno, 1997). In glioblastoma cells, constitutive activation of the PI3K/AKT signaling cascade and reduced levels of p21 protein expression are common, leading to defective cell cycle progression (Yang et al., 2016). It has been shown that TBMS1 reduces cyclin B1, CDK1, and AKT phosphorylation levels and elevates the level of the cKd1 inhibitor p21, which inhibits DNA synthesis and induces G2/M phase block in glioblastoma cells by targeting the PI3K/AKT/p21 and p21/CDK1/cyclin B1 signaling cascades (Cao et al., 2020).

Apoptotic signaling pathways are divided into death receptor (exogenous) pathways and mitochondrial (intrinsic) pathways (Creagh, 2014). The Bcl-2 family, which includes anti-apoptotic components such as Bcl-2 and Bcl-xl, as well as pro-apoptotic components such as Bax, Bak, and Bad, is involved in the regulation of apoptosis through the mitochondrial pathway. Mitochondria-dependent signaling occurs through the cleavage of caspase-9, which subsequently activates downstream caspase-3, leading to the cleavage of various key cellular substrates, including ParP, thereby inducing apoptosis (Palmer et al., 2011). Previous studies have shown that TBMS1 prevents glioblastoma progression by triggering apoptosis through the PI3K/AKT-mediated Bcl-2 signaling pathway via TBMS1. *In vitro* experiments showed that after 24 h of TBMS1 action, U251 cells showed chromatin condensation, nuclear fragmentation, and formation of apoptotic vesicles.

**FIGURE 5:**
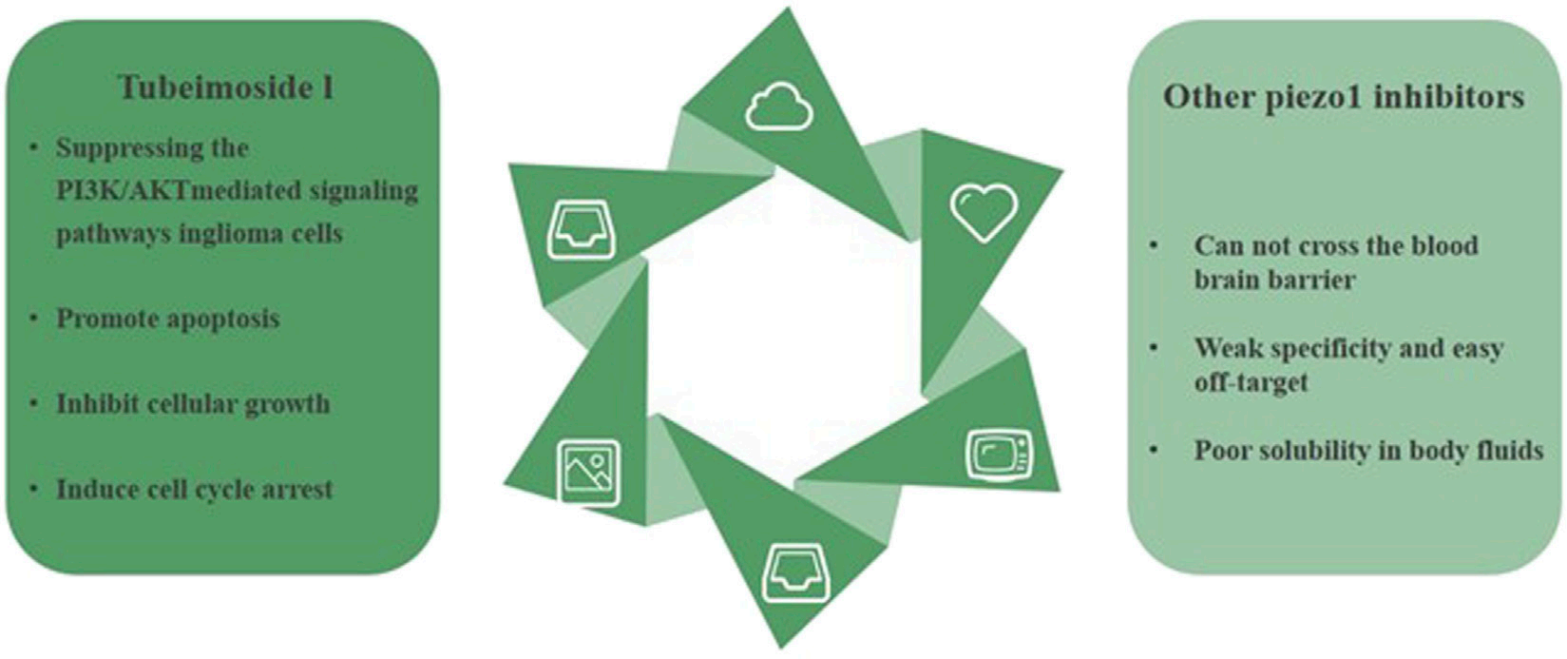
Tubeimoside I is currently the most rapidly studied Piezo1 inhibitor in the field of glioblastoma treatment. Compared to other inhibitors, Tubeimoside I has the advantages of stability, specificity, and high humoral solubility.

Overall, TBMS1 exhibited inhibition of cell growth, promotion of apoptosis, and induction of cell cycle arrest by inhibiting the PI3K/AKT-mediated signaling pathway in glioblastoma cells. This provides a theoretical basis for *in vivo* studies using TBMS1 as a potential cancer therapeutic compound (Cao et al., 2020). (Figure 5).

Although a variety of piezo1 inhibitors have been shown to inhibit tumor cell proliferation and promote apoptosis, most of the piezo1 inhibitors are non-specific inhibitors of piezo1 and have strong off-target properties. However, most of the piezo1 inhibitors are non-specific inhibitors of piezo1, with strong off-target properties, and most of the piezo1 inhibitors, except Tubeimoside I and GsMTx4, are unable to pass the blood-brain barrier, and they cannot act directly on glioblastoma tumor tissues, so their therapeutic application value is limited. At the same time, some inhibitors such as Dooku1 have poor solubility in body fluids, which seriously affects their activity *in vivo*.

## 6 Ultrasound activation of piezo1 for immunotherapy of glioblastoma

Studies have shown that it is feasible to treat glioblastoma with autologous T cells. However, the off-target effect due to the tumor microenvironment, tumor heterogeneity, and loss of antigenicity of glioblastoma makes accurate killing of glioblastoma difficult (Bagley et al., 2018). We can try to achieve controlled, non-invasive, and precise immunotherapy for glioblastomas by expressing Piezo1 on T cells and then locally stimulating glioblastomas using ultrasound or drugs targeting Piezo1, which activates Piezo1 and induces the activation of nuclear factor of t-cells (NFAT), which in turn drives the expression properties of the target genes (Pan et al., 2018).

Ultrasound and its associated energy can be delivered safely and non-invasively with high spatial and temporal resolution to small volumes of tissue deep within the body (Song et al., 2022; Zhu et al., 2023). Indeed, high-frequency ultrasound (HFU) can be focused for mechanical stimulation of individual cells in subcellular regions smaller than 10 μm. Due to the large difference in acoustic impedance between the surrounding medium and the air inside the bubble, microbubbles can further amplify the effects of low-frequency ultrasound stimulation with the ability to penetrate cells physically coupled to the microbubbles over long distances. The ultrasound power was stabilized in a range between 22.1 and 31.6 V to generate sufficient ultrasound to mechanically stimulate the cells without causing damage to the target cells.

**FIGURE 6:**
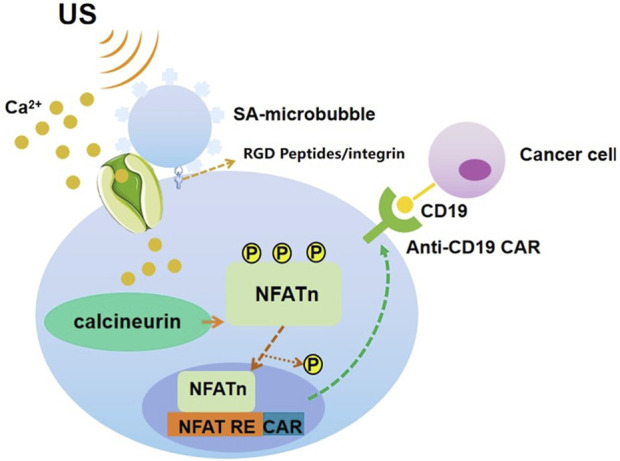
Schematic drawing of ultrasound-induced cell activation and gene expression. Microbubbles can be coupled to the surface of a cell, where mechanosensitive Piezo1 channels are expressed. Upon exposure to ultrasound waves, the mechanical stimulation can activate the Piezo1 ion channels. The subsequent calcium entry triggers the downstream pathways, including calcineurin activation, NFAT dephosphorylation, and translocation into the nucleus. The nucleus-translocated NFAT can bind to upstream response elements to initiate gene expression through one-stage or two-stage genetic transducing modules.

Ultrasound can be used to stimulate Piezo1 (Qiu et al., 2019) and subsequent calcium inward flow using microbubble coupling to activate the calcium-sensitive phosphatase calmodulin phosphatase, which dephosphorylates the nuclear factor of activated t-cells (NFAT), and then this transcription factor translocates to the nucleus to activate the NFAT response element (RE) to drive the expression of the designed target gene. Microbubbles mechanically coupled to Piezo1 can be used to amplify low-frequency 2 MHz ultrasonic mechanical waves over a centimeter distance. The microbubbles are coated with streptavidin and coupled to a biotinylated Arg-Gly-Asp (RGD) peptide, which binds to the integrin and connects to Piezo1 via the cytoskeleton and membrane tension to promote calcium influx into the cell upon ultrasound stimulation at 2 MHz. Jurkat T cells and monocytes implanted with microbubbles showed a significant increase in anti-CD19 CAR-encoding mRNA and expression in response to ultrasound stimulation. After co-incubation of the sonication-induced CAR-expressing Jurkat cells with target tumor cells expressing CD19 antigen for 24 h, the surface marker CD69, which reflects t-cell activation, was significantly upregulated in the Jurkat cells, indicating that sonication-induced CAR is produced in Jurkat T cells and mediates binding to target tumor cell antigens, thereby exerting a tumor cell killing effect (Pan et al., 2018). (Figure 6).

The system is highly modular, as shown by the multiple designs of genetic circuits with various base levels and activation potentials, thus allowing for continuous evolution and optimization for multiple types of cancer and pre-cancer diseases, and will bring the full power of remote control of gene and cellular activation to the scientific and clinical communities, thus allowing precise control of therapies in space and time, and ushering in a new era of life sciences and medical technologies.

## 7 Piezo1 as a target in combination with standard therapies for glioblastoma

### 7.1 Combined with immunotherapy and radiotherapy

Dendritic cells (DCs) are specialized antigen-presenting cells that play a crucial role in initiating immune responses to cancer immunotherapy through various activation pathways. Cell packets (BPs) are soft disk-shaped particles that modulate the phenotype of macrophages *in vivo*. The backpacks are prepared from biodegradable polymers using microcontact printing, and each backpack contains a cell adhesion layer, a poly (lactic-hydroxyglycolic acid) (PLGA) layer, a poly (vinyl alcohol) (PVA) layer and a second PLGA layer. Studies have shown that the “backpacks” can evade phagocytosis by macrophages, which enhances the stability of the drug *in vivo* (Shields et al., 2020). BPs represent an alternative approach for targeted delivery of immune activators and immune cells, providing prolonged immune modulation and enhanced therapeutic effects. Empty BPs act as modulators of DC immune function by activating cell-surface piezo1. Upon binding to DCs, BPs open piezo1 mechanoionic channels by binding to DCs, induce Ca^2+^ inward currents, activate local FAK and intracellular cytoskeletal remodeling to increase cellular stiffness, participate in the PI3K/Akt/mTOR cascade signaling to enhance IRF3 phosphorylation, and promote type I interferon production, and this activation leads to the initiation of the innate immunity-associated PI3K/Akt/mTOR/type I interferon signaling pathway. Radiation therapy combined with DC-BP (BPs that bind to DC cells) had a significant activating effect on the expression of mature dendritic cells, migrating dendritic cells, and lectin receptor-associated DCs, inducing a potent anti-tumor effect. Meanwhile, radiation therapy combined with DC-BP also significantly activated cytotoxic T lymphocyte (CTLs) in peripheral blood mononuclear cell (PBMCs), and activated T cells exhibited enhanced reactivity, leading to increased production of cytokines such as interferon gamma (IFN-γ), tumor necrosis factor-α (TNF-α), and interleukin-6 (IL-6), which exhibited potent systemic antitumor effects. This approach holds great promise for synergistic radioimmunotherapy in cancer treatment, providing a multimodal therapeutic platform with great potential. The DC-BP vaccine combined with radiotherapy has been shown to significantly promote tumor shrinkage and to have the same killing effect on distant tumors in bladder cancer.

In the future, it could be tried to improve the local control rate and prolong the survival of glioblastoma patients who undergo surgical treatment with standard post-surgical adjuvant concurrent radiotherapy (total dose of 60Gy for more than 6 weeks with daily temozolomide, followed by 6 cycles of temozolomide maintenance therapy) with activation of piezo1 by the DC-BP vaccine, offering new possibilities for glioblastoma treatment (Yu et al., 2024).

### 7.2 Combined with TKIs

The epidermal growth factor receptor (EGFR) gene is a tyrosine kinase receptor located on chromosome 7p11-p13, participating in key signaling pathways responsible for the cell cycle, growth, proliferation, migration, and survival of tumor cells. Mutations or overexpression are closely related to the development of more aggressive malignant phenotypes, leading to treatment resistance and poor clinical outcomes. After binding to endogenous ligands, EGFR forms homodimers or heterodimers, thereby activating the intracellular tyrosine kinase (TK) domain and causing phosphorylation of specific tyrosine residues. Under the operation of phosphorylated tyrosine, downstream PI3K/Akt, Ras/Raf/MAPK, JAK/STAT, and other pathways are activated. These pathways are closely related to the proliferation, invasion, and angiogenesis of gliomas, ultimately leading to the malignant biological behavior of gliomas. Currently, pan-targeted anti-angiogenic tyrosine kinase inhibitors (TKIs) targeting EGFR mainly act on the extracellular domain of EGFR, blocking intracellular signaling pathways such as MAPK and PI3K in tumor cells to control tumor growth, which is the direction of anti-EGFR treatment for gliomas. However, most of the current results are still in phase II clinical trials, and there is no reliable phase III clinical data to prove their effectiveness (Westphal et al., 2015; Lombardi et al., 2019).

Piezo1 activates EGFR through a typical epidermal growth factor (EGF) activation alternative pathway. During the steady-state cell turnover process, EGF activates EGFR signaling through tyrosine autophosphorylation, receptor internalization, and cytoplasmic extracellular regulated protein kinases (ERK) activation. Piezo1-dependent EGFR signaling (such as ERK activation and activator protein 1 (AP-1)induction) requires EGFR internalization through clathrin-mediated endocytosis (CME) rather than its kinase activity or Y1173 autophosphorylation, indicating that the internalization of the epidermal growth factor receptor is the key to piezo1 activation-induced AP-1. Internalized EGFR has a pro-survival function in kinase-independent cancer cells and is not affected by current tyrosine kinase-targeted therapy. The combination of Piezo1 inhibitors and TKI drugs provides a new direction for future targeted therapy of glioblastoma (Pardo-Pastor and Rosenblatt, 2023).

### 7.3 Combined with chemotherapy

Tumor necrosis factor-related apoptosis-inducing ligand (TRAIL), a member of the tumor necrosis factor-alpha family, induces apoptosis in cancer cells through death receptor activation (Lincoln et al., 2018; Fulda and Debatin, 2005). This ligand is expressed on the surface of activated natural killer (NK) cells, macrophages, DC, and T cells and plays a role in the innate immune response (Ralff and El-Deiry, 2018; Shepard and Badley, 2009). TRAIL has been found to trigger apoptosis in many tumor cell types by binding to death receptors 4 and 5 (DR4/5)(Lee et al., 2014). This binding leads to trimerization of the receptor, which then binds intracellularly to the Fas-associated death domain (FADD) and caspase-8 to form the death-inducing signaling complex (DISC). TRAIL is of particular interest in cancer therapy because of its ability to induce apoptosis in tumor cells without harming normal cells. However, xenograft animal studies have also shown that the use of TRAIL alone to treat the primary tumor itself is rarely successful. In addition, approximately 50% of tumor cell lines tested exhibit some degree of TRAIL resistance, and glioblastomas are particularly resistant to TRAIL. Therefore, for TRAIL to be a viable treatment, cancer cells must be sensitized to TRAIL (Lincoln et al., 2018; Fulda and Debatin, 2005).

One way in which TRAIL sensitization occurs is through the fluid shear stress (FSS) experienced by circulating tumor cells (CTCs) during circulation. Activation of the mechanosensitive ion channel Piezo1 via the agonist Yoda1 induces TRAIL sensitization under static conditions, and *in vivo*, Yoda1 significantly increased apoptosis in prostate, breast, and colon cancer cell lines compared to TRAIL alone (Hope et al., 2019). It was demonstrated that the combination of Yoda1 and TRAIL resulted in decreased GBM cell viability and increased late-stage apoptosis. The combination of FSS + TRAIL treatment resulted in a significant decrease in U87 and LN18 cell viability. These findings further demonstrated the efficacy of promoting TRAIL-mediated apoptosis through Piezo1 activation.

Combined treatment with Yoda1 and TRAIL increased mitochondrial depolarisation in GBM cells. Piezo1 has been shown to sensitize PC3 prostate cancer cells to TRAIL via the intrinsic apoptotic pathway, where Ca^2+^ activates calpain via Piezo1, subsequently amplifying death-inducing signals in the TRAIL-mediated apoptotic pathway (Knoblauch et al., 2023). These pro-apoptotic proteases induce pore formation in the mitochondrial membrane, which leads to mitochondrial outer membrane permeabilization (MOMP). Ultimately, MOMP leads to the release of apoptotic proteins through the mitochondrial pore, resulting in multiple cell death pathways (Hope et al., 2019; Chipuk et al., 2006).

Bax is a component of the intrinsic apoptotic pathway that triggers MOMP, and Bax channel blockers (BCBs) reduce mitochondrial depolarisation in GBM cells. Calpeptin, a calpain inhibitor, is an important component of the Piezo1 pathway. Yoda1 and TRAIL treatment inhibits calpain leading to a significant reduction in mitochondrial depolarisation.

Temozolomide (TMZ) is the standard chemotherapy used for the treatment of GBM.U87 cells are sensitive to TMZ, whereas LN18 cells are resistant to the drug. Studies have shown that LN18 cells are more sensitive to Yoda1 + TRAIL treatment than to TMZ treatment. U87 cells with low levels of Piezo1 expression responded comparably to both treatments. The sensitivity of these GBM cells to Yoda1 + TRAIL treatment suggests that the therapy may be successful in targeting tumors that are particularly resistant to current standard treatments. Thus Yoda1 + TRAIL combination therapy is expected to be a potential GBM treatment (Knoblauch et al., 2023).

Studies have shown that the abnormal activation of the PI3K/Akt/mTOR/NF-κB signaling pathway induced by EGFR promotes the proliferation of glioma cells and is related to MGMT promoter methylation or expression, subsequently enhancing glioma drug resistance, and the efficacy of chemotherapy drugs such as TMZ is weakened due to drug resistance. This is the main obstacle in the treatment of GBM. Studies have shown that TBMS1 can reduce the expression of EGFR in MGMT + cell lines by inhibiting the PI3K/Akt/mTOR/NF-κB signaling pathway. The combination of TBMS1 and TMZ strongly induces apoptosis in glioblastoma cells, accompanied by the formation of strong DSB, γH2AX foci, and an increase in PARP cleavage, and reduces the expression of MGMT in TMZ-resistant GBM cells, thereby reducing DNA damage repair and synthetic functions and restoring the sensitivity of MGMT + cells to TMZ. This study provides a theoretical basis for the combined application of TMZ and TBMS1 as a potential chemotherapy for MGMT + GBM patients (Tang et al., 2022).

### 7.4 Combined with antiangiogenic therapy

Bevacizumab is a recombinant humanized immunoglobulin G1 (IgG1) monoclonal antibody that can bind to VEGF-A, inhibit its binding to VEGF receptor-2 (VEGFR-2), and subsequently inhibit the biological effects of VEGF, including affecting vascular permeability, proliferation, and the migration and survival of endothelial cells. The anti-angiogenic therapy represented by Bevacizumab normalizes tumor vessels, leading to excessive pruning of endothelial cells and perivascular cells, reduced vascular tortuosity, and decreased interstitial pressure, thereby improving oxygenation and delivering chemotherapy to tumor cells, amplifying the anti-tumor effect of chemotherapy. Meanwhile, Bevacizumab can bind to VEGF, preventing VEGF from binding to its receptors on the surface of endothelial cells, thereby pruning vessels, regulating vascular permeability, reducing brain edema caused by brain necrosis, and treating brain necrosis (Nayak et al., 2021). Piezo1 inhibitors can block VEGF activation caused by piezo1. In the future, they can be tried in combination with bevacizumab to reduce abnormal glioblastoma vessels and inhibit tumor progression.

In addition to this, the piezo1 agonist Yoda1 + TRAIL can successfully kill glioblastoma cells that are particularly resistant to current standard therapies by activating piezo1, providing a new idea for the treatment of glioblastoma resistant to temozolomide.

## 8 Discussion

**FIGURE 7:**
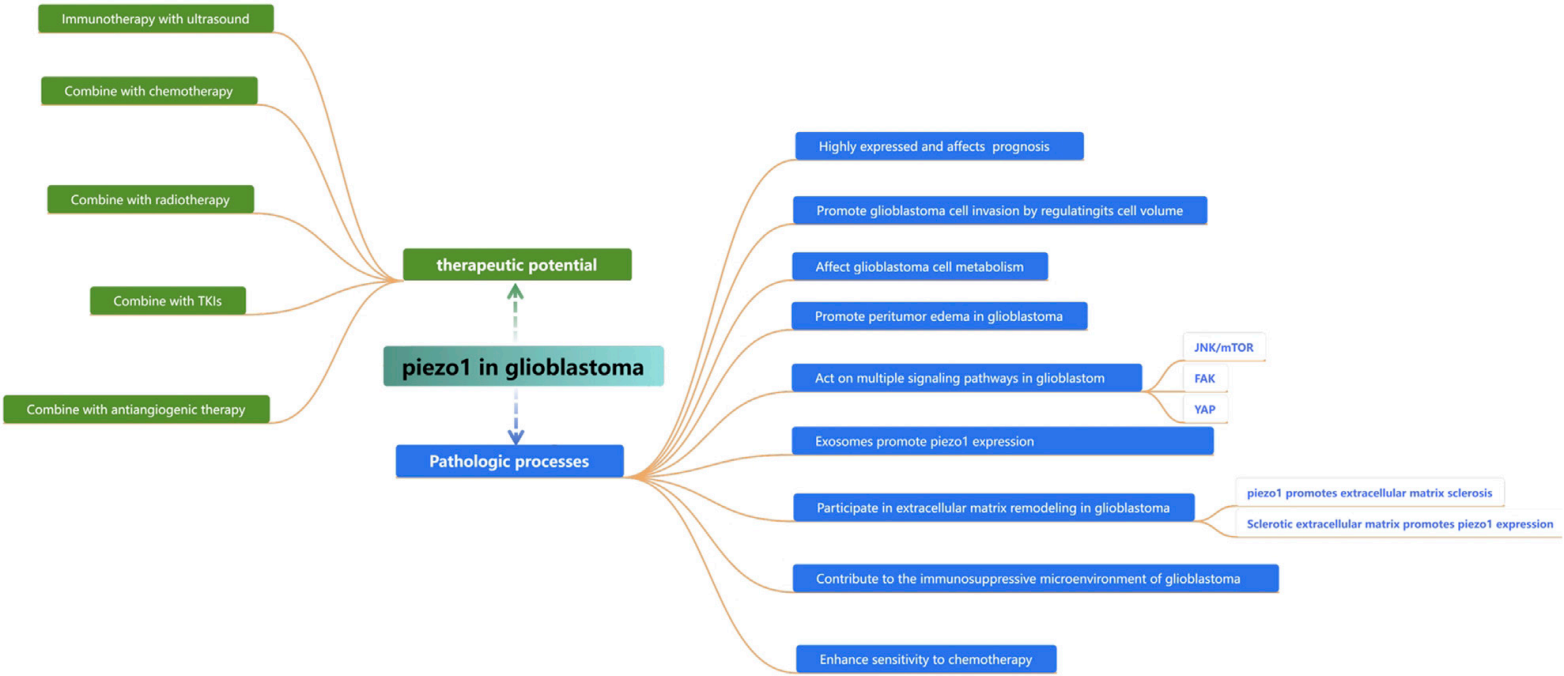
Summarizing the role of piezo1 in the proliferation, invasion, and progression of glioblastoma and its therapeutic potential.

Piezo1 is the first mechanosensitive cation channel protein identified and plays an important role in the development of a wide range of tumors. Glioblastoma, one of the most common and malignant brain tumors in adults, exhibits Piezo1 overexpression. Several studies have demonstrated that Piezo1 affects several underlying pathophysiological processes such as tissue hardening, angiogenesis, energy supply, and immune cell infiltration in glioblastoma, and can be used as an indicator of malignancy and prognostic assessment of glioblastoma patients, as well as a therapeutic target for controlling tumor progression (Figure 7). Specific mechanistic studies centered on Piezo1 will contribute to our understanding of the mechanistic biology of glioblastoma and help us develop new therapeutic approaches for glioblastoma patients.

## Glossary

GBM: glioblastoma
LGG and HGG: low to high-grade glioblastomas
ECM: extracellular matrix
ATP: adenosine triphosphate
CED: c-terminal extracellular structural domain
TM: transmembrane
OH - IH: outer helix-inner helix
CTD: c-terminal structural domain
THUs: transmembrane helical units
CSK: cytoskeleton
EMT: epithelial-mesenchymal transition
VEGF: vascular endothelial growth factor
VEGFR2: vascular endothelial growth factor receptor 2
WHO: World Health Organization
IDH: isocitrate dehydrogenase
G-CIMP: CpG island methylation
DNA: deoxyribonucleic acid
TSS: transcriptional start site
mRNA: messenger ribonucleic acid
OS: overall survival
shRNA: short hairpin RNA
ROS: reactive oxygen species
SDT: sonodynamic therapy
MCU: mitochondrial calcium uniporter
MRI: magnetic resonance imaging
PTBE: peritumoral brain oedema
SOCE: store-manipulated calcium endocytosis
NFAHFUT: nuclear factor of activated T cells
FAK: focal adhesion kinase
YAP: yes-associated protein
TAZ: transcriptional coactivator with PDZ-binding motif
miRNAs: micro RNAs
EVs: extracellular vesicles
3’UTR: 3‘ untranslated region
MMP: matrix metalloproteinase
TIMP: tissue inhibitor of metalloproteinases
MAPK: mitogen-activated protein kinase
PI3K: phosphoinositide 3 kinase
GAM: glioblastoma-associated microglia
TME: tumor micro-environment
GPD2: glycerol-3-phosphate dehydrogenase 2
PDGFR: platelet-derived growth factor receptor
JAK/STAT3: the Janus kinase 2/signal transducer and activator of transcription 3
BMDM: bone marrow-derived macrophages
RE: response element
EGF: epidermal growth factor
bFGF: basic fibroblast growth factor
IGF-1: insulin-like growth factor-1
SDF-1α: stromal-derived factor-1α
NF-κB: nuclear factor κ b
cAMP: cyclic adenosine monophosphate
CREB: -response element binding protein
MLCK: myosin light chain kinase
eNOS: endothelial-type nitric oxide synthase
ERK 1/2: extracellular signal-regulated kinase 12
TRAIL: tumor necrosis factor-related apoptosis-inducing ligand
NK: natural killer
DR4/5: death receptors 4 and 5
FADD: fas-associated death domain
DISC: death-inducing signaling complex
FSS: fluid shear stress
CTCs: circulating tumor cells
MOMP: membrane permeabilization
BCBs: Bax channel blockers
TMZ: temozolomide
RR: ruthenium red
Gd3+: Gadolinium
aβ: Amyloid
PUFAs: polyunsaturated fatty acids
AA: arachidonic acid
RVD: regulated volume reduction
VRAC: volume-regulated anion channel
ICl: swelling-activated CI − currents
KCa: Ca2+-activated K+
MSC: mechanosensitive channels
BK, IK, and SK: Ca2+-activated K+ large-, medium-, and small-conductance channels
JNK1: c-JunN-terminalkinase 1
mTOR: mammalian target of rapamycin
CDK4: cyclin-dependent kinases 4
cJUN: c-Jun N-terminal kinase
BAX: Bcl2-associated X
BAPTA-AM: 1,2-bis (2-aminophenoxy)ethane-n,n,n0,n0-tetraacetic Acid Tetrakis (acetoxymethyl Ester)
TRPV4: transient receptor potential ion channel subfamily V 4
TRP: transient receptor potential
ROCK: Rho-associated coiled-coil kinases
Treg: immunosuppressive regulatory T
RGD: biotinylated Arg-Gly-Asp
HFU: high-frequency ultrasound
DCs: dendritic cells
BPs: cell packets
CTLs: cytotoxic T lymphocyte
PBMCs: peripheral blood mononuclear cell
IFN: -γinterferon gamma
TNF-α: tumor necrosis factor-α
IL-6: interleukin-6
CXCL: C-X-C motif chemokine ligand
AVD: apoptotic volume decrease
MDSC: myeloid-derived suppressor cells
TAM: tumor-associated macrophages
CAF: cancer-associated fibroblasts
CRISPR: clustered regularly interspaced shortpalindromic repeats
Cas: -associated systems
MAPK: mitogen-activated protein kinase
Ras: rat sarcoma
Crk: cysteine-rich receptor-like protein kinases
Grb2: growth factor receptor-bound protein 2
LPS: lipopolysaccharide
HIF-1α: hypoxia-inducible factor 1α
PLGA: poly (lactic-hydroxyglycolic acid)
PVA: poly (vinyl alcohol)
DC-BP: BPs that bind to DC cells
EGFR: epidermal growth factor receptor
TK: tyrosine kinase
TKIs: tyrosine kinase inhibitors
EGF: epidermal growth factor
ERK: extracellular regulated protein kinases
AP-1: activator protein 1
CME: clathrin-mediated endocytosis
MGMT: O6 -methylguanine-DNA methyltransferase
IgG1: immunoglobulin G1
